# The effect of a fireworks event on the amount and elemental concentration of deposited dust collected in the city of Debrecen, Hungary

**DOI:** 10.1007/s11869-014-0290-7

**Published:** 2014-08-27

**Authors:** Edina Baranyai, Edina Simon, Mihály Braun, Béla Tóthmérész, József Posta, István Fábián

**Affiliations:** 1 Department of Inorganic and Analytical Chemistry, University of Debrecen, Debrecen, P.O. Box 21, 4010 Hungary; 2 Agilent Atomic Spectroscopy Partner Laboratory, Department of Inorganic and Analytical Chemistry, University of Debrecen, Debrecen, P.O. Box 21, 4010 Hungary; 3 Department of Ecology, University of Debrecen, Debrecen, P.O. Box 71, 4010 Hungary; 4 Hertelendi Laboratory of Environmental Studies, Institute of Nuclear Research of the Hungarian Academy of Sciences, Debrecen, 4026 Hungary; 5 MTA-DE Biodiversity and Ecosystem Services Research Group, Debrecen, P.O. Box 71, 4010 Hungary

**Keywords:** Fireworks show, Urban dust pollution, Elemental analysis, ICP-OES, MP-AES

## Abstract

Many social celebrations in urban areas are followed by fireworks show. The organic and inorganic pollutants emitted during detonations are expected to affect the ambient air quality of these celebration sites. The environmental aspects of fireworks events are usually investigated by analyzing the concentration and composition of airborne particulate matter, while there is limited information regarding the effect of fireworks on the elemental concentration of deposited dust. In this study, foliage dust samples were collected in the city of Debrecen (Hungary) before and after the fireworks show, organized on the 20th of August for the celebration of a historical event. Leaf samples (*Tilia tomentosa*) were collected around the location of the area of festivities. The sampling sites were further divided into five areas: city center (center), Southeast (SE), Southwest (SW), Northeast (NE), and Northwest (NW). We found that the amount of deposited dust particles increased significantly after the fireworks show compared to the background; we also found significant differences in the amount of dust deposition between the different locations of the city. A statistically higher level of Ca, Mg, and Sr was detected in samples collected after the display compared to those collected during the previous days, while the concentration of other studied elements were not statistically different from the background level. Our study confirmed previous findings that the relatively high altitude of detonations allows chemicals to disperse in the fine and ultrafine aerosol fractions; thus, the emitted pollutants by fireworks shows do not increase the level of elements as markedly in deposited dust as in the inhalable fraction.

## Introduction

Explosive pyrotechnic devices are widely used for celebrating specific events causing an unusual environmental effect on the ambient air quality (Vecchi et al. 2008). They are most frequently used in the already polluted urban areas (Kulshrestha et al. 2004), emitting additional amount of metal particles, gases, and various organic compounds which can cause a temporary decrease in air quality (Ravindra et al. 2003). The elemental concentrations of the aerosol particles that reach the atmosphere during fireworks events were studied using different analytical methods (Dutcher et al. 1999; Liu et al. 1997; Drewnick et al. 2005). It was demonstrated that the particulate matter generated by these shows contain Sr, K, V, Ti, Ba, Cu, Pb, Mg, Al, S, Mn, and Zn as major components (Perry 1999). It was found by Kulshrestha et al. (2004) that the concentration of Ba, K, Al, and Sr significantly increased during the fireworks of Diwali festival (India) compared to the background values registered on the previous days. This finding was confirmed by Sarkar et al. (2010). Most of these metallic salts serve as color-generating components in pyrotechnic devices and have adverse health effects if inhaled (Murty 2000; Hirai et al. 2000). Camilleri and Vella (2010) showed that most of the metals contributed by fireworks are in the inhalable dust—finding strong correlations between airborne particulate matter (PM10) and Al, Ba, Cu, Sr, and Sb concentrations in airborne dust—hence leading to a strong concern of health risks.

The environmental aspects of fireworks shows are usually studied by analyzing the concentration and composition of airborne particulate matter (PM10 and PM2.5) collected with conventional sampling methods (Guttikunda and Kopakka 2014; Rogula-Kozłowska et al. 2014; Wang et al. 2013; Tsai et al. 2012; Shen et al. 2009). There is no information in the literature on the deposited dust in urban areas from fireworks; in most of the studies, the fine and/or inhalable fractions were investigated due to health concerns. However, deposited dust may contain toxic substances affecting plants, the quality of soil, and groundwater especially in urban and rural areas (Pelig-Ba et al. 2001; Goossens 2007).

Trees reflect the cumulative effects of environmental pollution from both the soil and the atmosphere (Madejón et al. 2004). Therefore, they have been used as bioindicators in several studies (Celik et al. 2005; Baycu et al. 2006). Mostly plant tissues are analyzed to monitor the accumulation of trace elements confirming their availability in the soil (Baker et al. 2000; Markert et al. 2003) and atmosphere since they can capture and uptake trace elements as well as concentrate them, making small amounts detectable. However, trees can also be used as dust traps in environmental assessments based on urban dust analysis (Margitai and Braun 2005) since deposited contaminants can be trapped by the surface of leaves thus representing the environmental load. Although Simon et al. (2011) concluded that the elemental concentration of foliage dust did not differ remarkably between species, the amount of captured deposited dust highly depends on the surface of leaves, stomata size, and density of the selected species. Accordingly, these factors affect their possible use in air quality monitoring investigations (Abbruzzese et al. 2009).

Since leaf sampling is widely used in pollution studies due to the inherent variability in crowns (Luyssaert et al. 2002), our aim was to investigate the applicability of this dust sampling for the analysis of deposited dust load and trace element emission resulted by a fireworks event.

## Materials and methods

### Sampling procedure

Leaf samples were collected in the city of Debrecen the days before and after the fireworks show on 20th of August (2011), which commemorated a specific historical event in Hungary. Debrecen is the second largest city in Hungary with nearly 225,000 inhabitants. It is the regional center of the Northern Great Plain region and situated 220 km east of the capital, Budapest.

**Fig. 1:**
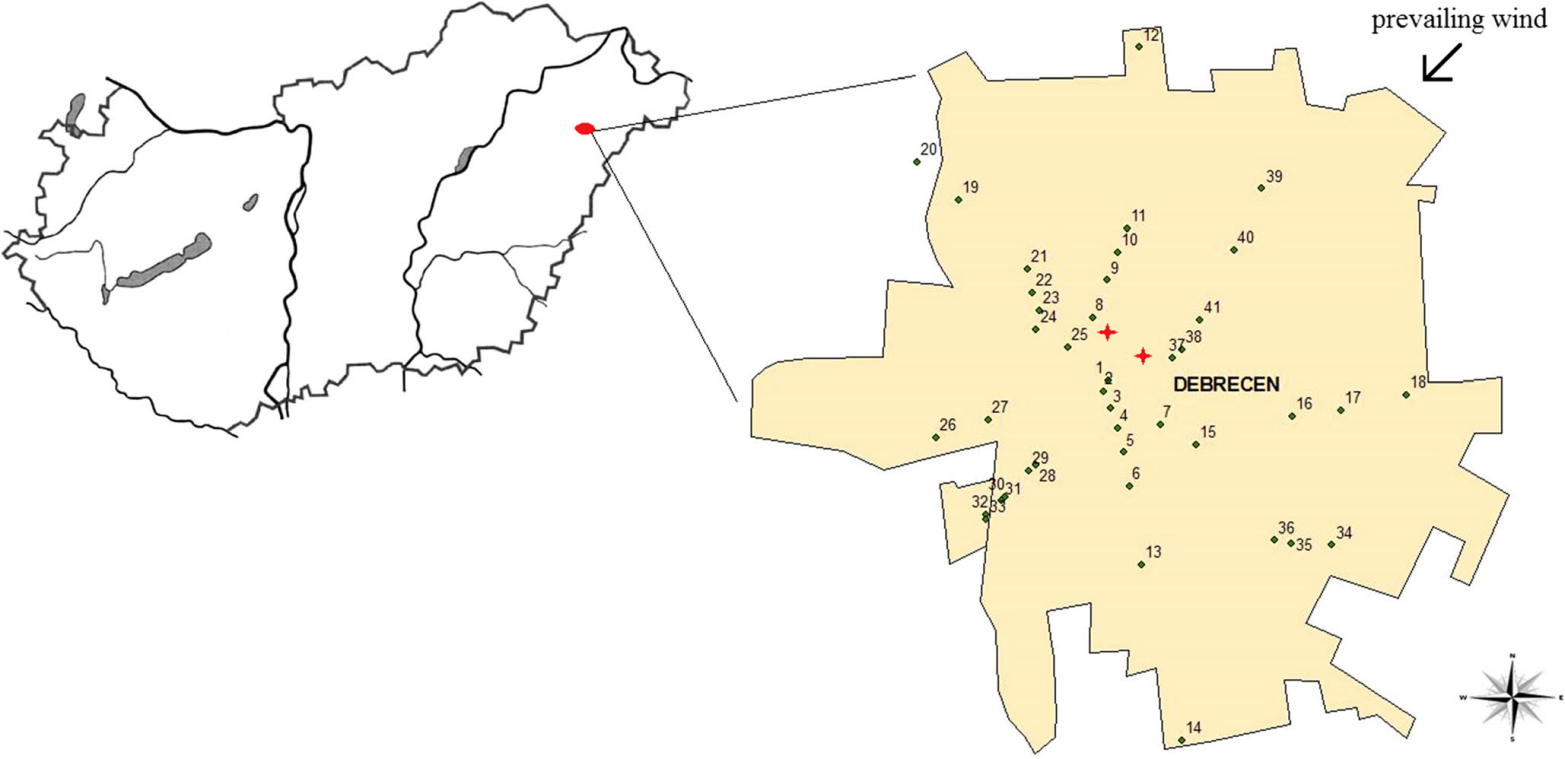
Locations of the 41 sampling sites and the fireworks events indicated on the city map of Debrecen

The fireworks event was organized simultaneously in two locations in the city center. We divided the sampling sites into five subareas as follows: city center (center), Southeast (SE), Southwest (SW), Northeast (NE) and Northwest (NW) (Fig. 1). Silver linden (*Tilia tomentosa*) leaves were chosen since the morphology, canopy structure, and the dust preserving epicuticular wax on the leaf surface make linden trees ideal for biomonitoring studies (Braun et al. 2007). These trees are widespread in and around the city of Debrecen and can be easily distinguished from other species. We collected the control samples 2 days before the festivities (18th and 19th of August, 2011) from the individual selected species; the trees were marked and their coordinates were registered. Sample collection from the same 41 individuals was repeated 2 days after the fireworks show (21st and 22nd of August, 2011). The sampling procedure is based on literature values (Moreno et al. 2010). The pyrotechnic event was performed during stable meteorological conditions, and the weather was also dry in the time of the two sampling periods. The prevailing wind of Debrecen is directed NW–SE (Lóki et al. 1993). The total leaf surface of each sampled tree represented 10–12 dm^2^. Samples were collected in paper bags, and they were stored at +4 °C for analysis.

### Sample preparation

The surface area of leaves was determined by a flat scanner. The foliar dust particles were washed down from leaves by deionized water obtained from a Millipore Synergy Ultrapure Water System. Leaves collected from the same individuals were placed into 500-ml plastic container and 250 ml of deionized water was added; then, samples were shaken in an ultrasonic bath (Elma Transsonic, 460/H) for 10 min. The dust containing suspension was filtered through a 150-μm sieve, and the procedure was repeated with 50 ml deionized water. This process produced 300 ml dust containing suspension which was transferred into a microwave digestion system (MLS 1200 mega) where its volume was reduced to 20–30 ml. Then, the suspension sample was transferred into 50-ml glass beaker without loss, and the rest of the water was evaporated at 105 °C in a drying cabinet. The beakers were reweighted to determine the dry weight of dust on an analytical balance (Precisa 240A). Samples were prepared prior to analysis in the same vessels by acid digestion using the mixture of 5 ml 65 % (m/m) nitric acid (reagent grade, Merck) and 2 ml 30 % (m/m) hydrogen-peroxide (reagent grade, Scharlau) on an electric hotplate at 80 °C for 4 h. Only those particles were chemically decomposed, which are not vaporized at temperatures up to 105 °C. Digested samples were diluted up to 10 ml using 1 % (m/m) nitric acid (Simon et al. 2011).

### Elemental analysis of dust samples

The determination of Al, As, Ba, B, Ca, Cu, Fe, K, Mg, Mn, Na, P, S, and Zn was carried out by inductively coupled plasma optical emission spectrometry (ICP-OES, IRIS Intrepid II XSP), while the concentration of Li and Sr was determined by microwave plasma atomic emission spectrometry (MP-AES 4100, Agilent Technologies). The certificated material used was ERM-CZ120. The recovery for all elements was under 5 %.

Six-point calibration solution series were diluted from multi-element calibration stock solution of 1,000 mg l^−1^ (Merck ICP multi-element standard solution IV).

### Statistical analysis

Data were log transformed prior to analyses. The distribution of data was tested with Shapiro–Wilk test. Principal component analysis (PCA) was used to display the effect of sampling period (before and after the fireworks event) as well as the sampling sites (center, SE, SW, NE, NW) on the concentration of the selected elements in the foliage dust. The homogeneity of variances was tested by Levene’s test. The effect of sampling locations on the amount of deposited dust on the surface of leaves and the elemental concentration of deposited dust was studied by general linear model (GLM). Tukey’s multiple comparison test was used to explore the significant differences. The *t* test was used to compare the amount and elemental concentration of deposited dust before and after the fireworks show.

## Results

### Amount of dust deposited on the surface of leaves

**Fig. 2:**
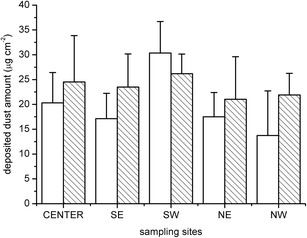
The amount of foliage dust before and after the fireworks show in terms of the sampling sites given in micrograms per square centimeter, (mean ± SD). *Open column* before fireworks, *hatched column* after fireworks. *SE* Southeast, *SW* Southwest, *NE* Northeast, *NW* Northwest

According to the *t* test, there was a significant difference (*t*_80_ = −2.178; *p* = 0.032) between the amount of dust deposited on the surface of the collected leaf samples before and after the fireworks show (Fig. 2). The foliage dust significantly increased based on the GLM results after the fireworks event in all studied areas except the Southwestern (SW) region of the city, where the amount of dust was significantly lower after the event. Sampling period and sampling area had a significant effect (period *F* = 5.274, *p* = 0.025; area *F* = 5.665, *p* = 0.001) on the amount of foliage dust while the interaction effect was not significant (*F* = 1.939; *p* = 0.113). The highest dust enlargement after the fireworks show occurred in the Northwestern region (NW) of the city. Considering the amount of deposited dust, the Southwestern area differed significantly from the Southeastern (*p* < 0.05), Northeastern (*p* < 0.05), and Northwestern (*p* < 0 0.001) areas of the city both before and after the fireworks show, but did not differ significantly from the center.

### Elemental concentration of foliage dust

The concentration of the measured elements in the foliage dust in terms of sampling time and area is indicated in Table 1. The GLM analysis showed significant differences between the sampling period (*F* = 4.794, *p* < 0.001) and among sampling area (*F* = 2.636, *p* = 0.002), based on the elemental concentration of deposited dust, while their interaction effect was not significant (*F* = 1.279; *p* = 0.112).

**Table 1:** Concentration of the measured elements given in micrograms per kilogram (mean ± SD) in foliage dust of sampling sites before and after the fireworks show

Elements (mg kg^−1^)	Center		SE		SW		NE		NW	
	Before	After	Before	After	Before	After	Before	After	Before	After
Al	4664 ± 1291	5023 ± 703	6753 ± 1649	6018 ± 1294	5250 ± 1851	5941 ± 805	4890 ± 905	5700 ± 1211	5955 ± 1112	6244 ± 1463
As	41 ± 181	5 ± 2	66 ± 16	10 ± 7	40 ± 24	8 ± 2	16 ± 7	10 ± 8	18 ± 9	19 ± 3
Ba	124 ± 53	126 ± 53	133 ± 41	114 ± 37	77 ± 16	89 ± 13	92 ± 15	102 ± 12	234 ± 164	262 ± 225
B	650 ± 897	924 ± 1822	819 ± 793	752 ± 805	510 ± 423	750 ± 736	963 ± 516	1251 ± 744	290 ± 305	420 ± 199
Ca	23870 ± 5908	27594 ± 6668	27808 ± 6694	25310 ± 6683	24874 ± 5282	34357 ± 5643	31179 ± 9899	40483 ± 4968	23419 ± 7123	37417 ± 6638
Cu	134 ± 97	151 ± 46	174 ± 125	126 ± 99	115 ± 73	100 ± 54	88 ± 12	88 ± 41	168 ± 56	159 ± 74
Fe	11024 ± 2478	12561 ± 2966	11281 ± 2275	11316 ± 2957	7543 ± 1923	9217 ± 1877	7605 ± 1524	9823 ± 2586	12101 ± 4220	11203 ± 4348
K	37334 ± 8892	34704 ± 12778	31408 ± 12185	22159 ± 13539	36482 ± 11436	40638 ± 16038	32215 ± 17175	29003 ± 18054	24581 ± 9681	26908 ± 11944
Li	14 ± 9	13 ± 12	20 ± 8	18 ± 8	19 ± 16	28 ± 33	61 ± 106	64 ± 116	14.2 ± 2.5	15 ± 7
Mg	5236 ± 1433	5870 ± 1694	6604 ± 1626	5734 ± 1756	6140 ± 1143	7124 ± 1400	9206 ± 5898	9845 ± 5323	4634 ± 783	7692 ± 727
Mn	333 ± 80	364 ± 73	458 ± 131	426 ± 99	375 ± 126	470 ± 133	404 ± 26	497 ± 68	421 ± 99	404 ± 91
Na	5906 ± 4439	5535 ± 5021	20304 ± 22431	17144 ± 18594	4654 ± 4159	6583 ± 8908	3504 ± 1625	3718 ± 1573	7243 ± 1473	8230 ± 8948
P	17657 ± 4033	15805 ± 3853	22105 ± 5117	15079 ± 3186	17382 ± 9690	19507 ± 4454	14433 ± 2212	16970 ± 4856	16933 ± 3301	21469 ± 6313
S	11809 ± 3687	10616 ± 3812	17450 ± 3567	9417 ± 2060	11815 ± 5913	10366 ± 2690	9089 ± 1081	9827 ± 1372	10959 ± 3756	14265 ± 2588
Sr	156 ± 50	201 ± 63	225 ± 57	194 ± 33	202 ± 72	224 ± 63	298 ± 155	325 ± 167	139 ± 46	213 ± 52
Zn	390 ± 110	334 ± 59	646 ± 315	346 ± 159	327 ± 189	288 ± 49	241 ± 70	277 ± 64	430 ± 138	425 ± 151

*Center* city center, *SE* Southeast, *SW* Southwest, *NE* Northeast, *NW* Northwest

#### Elemental concentration of foliage dust before the fireworks show

Significant differences occurred in the elemental concentration of foliage dust considering the sampling areas before the fireworks event. The concentration of Cu was the lowest (*p* < 0.001) in the Northeastern area of the city, while the concentration of Ba was the highest (*p* < 0.001) in the Northwestern area compared to the other studied locations. The centrum area differed significantly from the Southeastern area for Al, K, and Na (*p* < 0.05) and from the Northeastern are for Ca (*p* < 0.05) and Mg (*p* < 0.001). The highest Mg concentration was measured in the Northeastern area, showing a significant difference compared to the others. The Fe concentration was significantly lower (*p* < 0.05) in the Southwestern and Northeastern areas of the city compared to the other studied locations. The Southeastern area showed a statistical difference in the Zn (*p* < 0.001) and S (*p* < 0.05) concentrations compared to the Northeastern area.

#### Elemental concentration of foliage dust after the fireworks show

According to the *t* test, the level of Ca (*t*_80_ = −3.701, *p* < 0.001), Mg (*t*_80_ = −2.104, *p* = 0.039), and Sr (*t*_80_ = −2.292, *p* = 0.025) increased significantly in foliage dust after the fireworks event, while the concentration of As (*t*_80_ = 4.872, *p* < 0.001) statistically decreased.

The GLM results proved that the concentration of Ca, Mg, and Sr statistically increased in the centrum, Southwestern, Northeastern, and Northwestern sampling areas, respectively (*p* < 0.005). A higher level of Al, Ba, B, Cu, Mn, Li, Na, and P was also observed in the centrum, Southwestern, Northeastern, and Northwestern sampling areas, although the difference was not significant. None of the studied elements showed significant concentration increase in the Southeastern sampling area after the fireworks event.

**Fig. 3:**
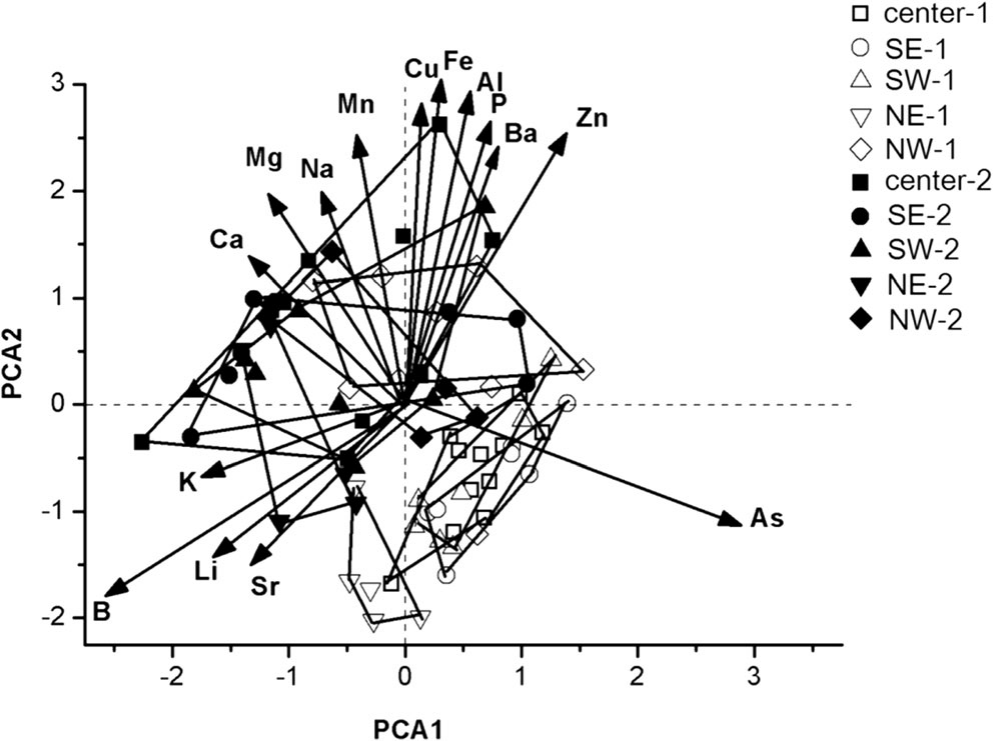
Principal component biplot of the measured elements in the foliage dust of the sampling sites before and after the fireworks show. *Center* city center, *SE* Southeast, *SW* Southwest, *NE* Northeast, *NW* Northwest. Numbers after the notations indicate samples collected before (*1*) and after (*2*) the fireworks event

According to PCA there is a clear separation of elements before and after the fireworks show as well as for the studied areas except for the center. The first component (PC1) contributed 32.9 % while the second (PC2) contributed 26.1 % to the total variance. As indicated in Fig. 3, positive correlation was found for Zn, Ba, and Al with PC1, while K, B, Li, and Sr correlated negatively with the same axis. Positive correlation occurred for Cu, Fe, Mn, Mg, and Ca, while negative correlation was found for Sr and As with PC2.

## Discussion

In the current study, the effect of a fireworks event is investigated by measuring the amount and elemental concentration of foliage dust deposited on the surface of silver linden tree leaves in the city of Debrecen. Earlier assessments of the effects of compounds emitted during fireworks events are quite controversial. It was stated by Perry (1999) that no significant health concern is involved in pyrotechnic displays. There are studies in the literature which challenge this statement and prove the negative effect of fireworks on the ambient air quality (Ravindra et al. 2003; Camilleri and Vella 2010). These shows may cause adverse health and environmental problems. Sarkar et al. (2010) investigated the amount of fine, respirable particles (PM10) during a major fireworks festival in India and concluded that the level of 24 h PM10 was extremely high. Moreno et al. (2010) analyzed the background level of ultrafine particles during a fireworks pollution episode in Girona (Spain) and observed a highly increased level of 24 h PM2.5. However, no information is found in the literature about the relation of fireworks displays and the amount of deposited dust. In our study, the overall amount of foliage dust was significantly higher after the fireworks compared to the background amount collected previously. Considering the sampling areas, the amount of deposited dust statistically increased after the event in all studied areas except the Southwestern part of the city. This phenomenon may be the consequence of the particular climate and relief of Debrecen. The prevailing wind of Debrecen is directed NW–SE, and in the Northwestern part of the city, a woodland is located protecting that part from strong wind blows (Lóki et al. 1993). On the contrary, the Southwestern region of the city is an open field district with the lack of continuous tree borders and with wind tunnel effect of high buildings. Probably, this is the reason for the decreased dust amount in the Southwestern sampling area.

During fireworks occasions, the concentration of elements such as Sr, Mg, K, Ba, and Cu is reported to be remarkably higher in airborne dust (Wang et al. 2007; Moreno et al. 2010) indicating the presence of highly toxic metals in fireworks devices. In the current study, the level of Ca, Mg, and Sr increased statistically. These elements are used as color-generating agents in pyrotechnic devices. We found no significant increase in the concentrations of Al, Ba, B, Cu, Mn, Li, and Na elements after the fireworks event, and their concentrations after the fireworks show are far below than reported by other research groups (Ravindra et al. 2003). These results are in contrast to our previous hypothesis according to which we would expect high emission of these toxic elements after the fireworks event. Yet, this finding is very similar to what Vecchi et al. (2008) concluded. They found significantly higher elemental concentration in PM10 fraction but they detected no increase in the course fraction of dust particles: the level of Sr, Mg, K, Ba, and Cu was below or comparable after the fireworks event in the coarse fraction of collected aerosol particles during a fireworks episode in Milan. They concluded that the ambient aerosol after the fireworks event was preferably confined in the fine fraction (Vecchi et al. 2008). Crespo et al. (2012) gained very similar findings. They discussed that the firework-related metals were concentrated in the submicrometric region (>80 %) of the collected dust particles. Our study proves these statements since only Ca, Mg, and Sr were in significantly higher concentrations; other differences were not detected in toxic metal concentrations in deposited dust after the fireworks show. Therefore, we further conclude that pyrotechnically derived aerosol particles containing inorganic pollutants are in the fine fraction, while the heavy fraction of dust particles depositing after the show did not pose environmental risk with respect to toxic elements.

The background elemental concentration of foliage dust showed differences between the studied areas. The Southeastern part of the city is the most contaminated considering the measured elements, where Al, Cu, Mn, Na, P, and Zn is in the highest concentration. Mingorance and Oliva (2006) concluded that the accumulation of toxic elements in leaf samples depends on the urbanization levels. The Southeastern part of Debrecen represents the highest level of urbanization, where the Debrecen Airport and also an industrial area are located with heavy traffic load, which explain the statistically higher amount of pollutants in foliage dust. The Southwestern and centrum areas proved to be the less contaminated with the measured elements based on the foliage dust analysis. The centrum area is in the city center, where a long pedestrian street is located with nearly no traffic load. The Southwestern part of Debrecen is characterized by a green belt with garden houses and small open-space parks, making this area less affected by human activities. The K concentration was the highest in these two sampling areas, which is in good agreement with Simon et al.’s (2011) previous findings. They analyzed foliage dust and leaf samples in and around the city of Vienna (Austria) along an urbanization gradient and measured a significantly higher K level in the rural sampling area, which represented an undisturbed part of the city with decreased human impact. Beddows et al. (2004) stated that potassium is a crustal component and as a major trace element plays an important role in plant grow hence it occurs frequently in rural areas. It was also found by Beddows et al. (2004) that 87 % of coarse particles of rural background atmospheric aerosol collected in the UK contained potassium. The origin of the higher K concentration in the Southwestern region may be from the biomass burning as there is a power plant located near that part of the city.

## Conclusions

The fireworks show in Debrecen resulted in a higher amount of dust particles deposited on the surface of tree leaves. Our findings suggest that the relatively high altitude of detonations allow the chemicals to disperse in the fine and ultrafine fractions. Thus, emitted pollutants by firework displays did not elevate the concentration levels of elements in deposited dust since the background concentration of these inorganic components in heavy dust is already high in polluted cities like Debrecen. Pyrotechnic detonations therefore cause environmental risk by the elevated amount of dust deposition but do not contribute significantly to the background concentration of toxic metals in foliage dust.
